# Probiotics Modulate Tilapia Resistance and Immune Response against Tilapia Lake Virus Infection

**DOI:** 10.3390/pathogens9110919

**Published:** 2020-11-06

**Authors:** Pitchaporn Waiyamitra, Mehmet Arif Zoral, Aksorn Saengtienchai, Amorn Luengnaruemitchai, Olivier Decamp, Bartolomeo Gorgoglione, Win Surachetpong

**Affiliations:** 1 Graduate Program in Animal Health and Biomedical Sciences, Faculty of Veterinary Medicine, Kasetsart University, Bangkok 10900, Thailand; pitchaporn.waiy@ku.th; 2 Department of Veterinary Microbiology and Immunology, Faculty of Veterinary Medicine, Kasetsart University, Bangkok 10900, Thailand; 3 Aquatic Animal Health Laboratory, Dept. of Pathobiology and Diagnostic Investigation, College of Veterinary Medicine—Michigan State University, East Lansing, MI 48824, USA; zoralmeh@msu.edu (M.A.Z.); BartGorg@msu.edu (B.G.); 4 Aquatic Animal Health Laboratory, Dept. of Fisheries and Wildlife, College of Agriculture and Natural Resources—Michigan State University, East Lansing, MI 48824, USA; 5 Department of Pharmacology, Faculty of Veterinary Medicine, Kasetsart University, Bangkok 10900, Thailand; fvetasc@ku.ac.th; 6 Manit Genetic Co Ltd., Phetchaburi 76140, Thailand; amorn.l@manitfarm.com; 7 Inve Technologies, 9200 Dendermonde, Belgium; o.decamp@inveaquaculture.com

**Keywords:** aquaculture, red hybrid tilapia, *Oreochromis* spp., antiviral response, *Bacillus* spp., *Tilapia tilapinevirus*, TiLV, immunity

## Abstract

Tilapia lake virus (TiLV) causes an emerging viral disease associated with high mortality and economic damage in tilapia farming around the world. The use of probiotics in aquaculture has been suggested as an alternative to antibiotics and drugs to reduce the negative impact of bacterial and viral infections. In this study, we investigate the effect of probiotic *Bacillus* spp. supplementation on mortality, viral load, and expression of immune-related genes in red hybrid tilapia (*Oreochromis* spp.) upon TiLV infection. Fish were divided into three groups, and fed with: control diet, 0.5% probiotics-supplemented diet, and 1% probiotics-supplemented diet. After 21 days of experimental feeding, the three groups were infected with TiLV and monitored for mortality and growth performances, while organs were sampled at different time points to measure viral load and the transcription modulation of immune response markers. No significant difference was found among the groups in terms of weight gain (WG), average daily gain (ADG), feed efficiency (FE), or feed conversion ratio (FCR). A lower cumulative mortality was retrieved from fish fed 0.5% and 1% probiotics (25% and 24%, respectively), compared to the control group (32%). Moreover, fish fed with 1% probiotic diet had a significantly lower viral load, than those fed with 0.5% probiotic and control diet at 5, 6, 9, and 12 days post infection-challenge (dpc). The expression patterns of immune-related genes, including *il-8* (also known as CXCL8), *ifn-γ*, *irf-3*, *mx*, *rsad-2* (also known as VIPERIN) showed significant upregulation upon probiotic treatment during the peak of TiLV pathogenesis (between 9 and 12 dpc) and during most of the study period in fish fed with 1% probiotics-supplemented diet. Taken together, these findings indicate that dietary supplementation using *Bacillus* spp. probiotics may have beneficial effects to strengthen tilapia immunity and resistance against TiLV infections. Therefore, probiotic treatments may be preventively administered to reduce losses caused by this emerging viral infection in tilapia aquaculture.

## 1. Introduction

Tilapia are the most economically important farmed fish species produced in aquaculture systems worldwide [1]. In 2018, global tilapia production was estimated at 6.8 million tonnes, accounting for an economic value of USD 11 billion [2]. Although tilapia can adapt and tolerates variable farming conditions, recent outbreaks of emerging bacterial and viral diseases pose severe threats to the global tilapia aquaculture production [3]. Recently, the outbreak of a new viral disease, caused by tilapia lake virus (TiLV), has drawn attention due to the rapidity of it spreading between farms and countries [4,5]. TiLV was identified as a single-stranded, negative-sense RNA virus sharing some characteristics with other viruses in the family Orthomyxoviridae [6,7]. Later, the virus was classified as a new species, *Tilapia tilapinevirus*, under the genus *Tilapinevirus*, but in the family Amnoonviridae [8]. Mass mortality of tilapia associated with TiLV is reported in many countries, such as North America, South America, Asia, and Africa [9,10]. All stages of tilapia farming including fry, juveniles, adults, and broodstocks are susceptible to TiLV infection, with a wide range of morbidity and mortality ranging from 5 to 90% [7,10,11,12,13,14,15]. As there is no specific treatment against TiLV infections, preventing or reducing the infection risk is an important control measure to minimize the negative impact of this infection once the virus is expected to enter a production system.

The application of probiotics to reduce bacterial and viral infections in fish is seen as an alternative strategy to improve farmed fish health. Multiple bacterial species have been used to formulate probiotics for aquaculture, including a wide range of Gram-positive, Gram-negative bacteria, or yeasts, such as *Bacillus*, *Carnobacterium*, *Enterococcus*, *Lactobacillus*, *Vibrio*, *Pseudomonas*, and *Saccharomyces cerevisiae* [16,17,18]. Among these, bacteria of the genus *Bacillus* are commonly applied as feed additives due to their potential benefits for improving growth [19,20,21], promoting disease resistance [22] and boosting the host immune response [23]. In tilapia, *Bacillus* extracts probiotics were shown to improve resistance against *Streptococcus agalactiae* and *Aeromonas hydrophila*, two common and important bacteria that pose health issues to the global tilapia aquaculture [24,25]. Although probiotics have been commonly used in aquaculture to control bacterial infections, previous studies also indicated the benefits of probiotic supplementation in farmed fish diets to improve growth performance [26] and resistance to virus infections [27,28]. For example, olive flounder (*Paralichthys olivaceus*) fed with Lactobacil and Sporolac (Inter Care, India) probiotics showed higher survival against lymphocystic disease virus (LCDV) [27]. Similarly, orange-spotted grouper (*Epinephelus coioides*) fed a probiotics-supplemented diet for 28 days had over 50% survival during grouper iridovirus (GIV) infection than fish fed the control diet [29].

Currently, there are no control measures or vaccines available against TiLV infection in tilapia. The aim of this study was to evaluate the effects of *Bacillus* spp. probiotics supplementation to improve the survival of tilapia during TiLV infection. Our results reveal that such dietary supplementation could improve fish survival and strengthen the antiviral response upon TiLV infection. Therefore, the administration of *Bacillus* spp. probiotics could provide a suitable alternative strategy to mitigate the losses caused to tilapia farming by this emerging viral threat.

## 2. Results

### 2.1. Bacillus spp. Probiotics Improve Tilapia Survival against TiLV Infection

**Figure 1:**
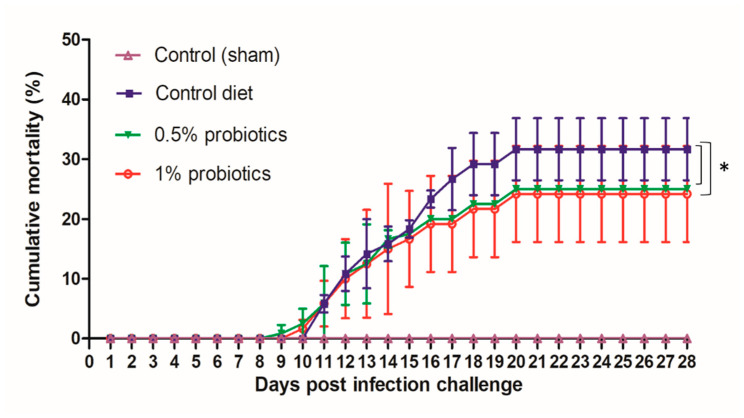
Cumulative mortality of red hybrid tilapia fed with the standard control diet, or with diets supplemented with 0.5% or 1% of a commercial mixture of *Bacillus* spp. Fish were challenged by cohabitation with tilapia lake virus-infected fish. Each group has 3 replicates with 40 fish/tank. All treatment groups were compared to the control diet group with significant differences shown as: * *p* < 0.05.

During our infection challenge study, TiLV infection was obtained upon cohabitation of TiLV-IP injected red tilapia with fish fed control or probiotic diets. At 3 days post infection-challenge (dpc), the IP-injected fish started to develop typical TiLV infection symptoms, including lethargy (lying at the bottom of the tanks), and haemorrhaging along the body side and at the base of the fins [30]. As the pathogenesis progressed, the mortality of IP-injected fish started at 5 dpc, reaching a cumulative mortality of 96.57% (84.62%–100%) (experiment terminated at 28 dpc). All probiotics and control diets fed red tilapia that cohabitate with IP-injected fish showed similar clinical signs of TiLV infection, starting at 7 dpc. Furthermore, mortality in the cohabitated groups started at 9 dpc, and persisted until 20 dpc (Figure 1). The cumulative mortality reached in the control group was 32%, while in groups preventively fed with 0.5% and 1% probiotics-supplemented diets, was, respectively, of 25 and 24%. Both probiotics treatments significantly improved the survival of red tilapia over TiLV infection (*p* < 0.05) (Figure 1). No mortality or clinical signs were observed in the sham-challenged group during the entire study. The growth performance parameters assessed, including weight gain, average daily gain, feed conversion ratio, and feed efficiency were not significantly changed in all experimental groups (Table 1). However, although the 1% *Bacillus* spp.-supplemented diet group showed the greatest weight gain and feed conversion ratio by the end of the experiment, there was no statistical significance of these values between the control and probiotics supplementation groups.

### 2.2. Differential Viral Load Assessment

**Figure 2:**
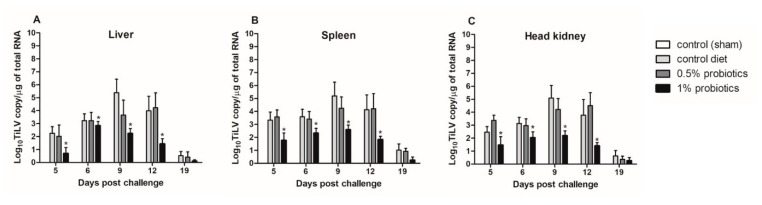
Viral load in liver (**A**), spleen (**B**) and head kidney (**C**) of red hybrid tilapia (*n* = 5) fed control or *Bacillus* spp. probiotics-supplemented diets for 21 days and thereafter infected with TiLV. Data were collected from five fish at 5, 6, 9, 12 and 19 dpc. All treatment groups were compared to the control diet group with significant differences shown as: * *p* < 0.05.

Although TiLV clinical signs were still not apparent in the cohabitation group when the first IP-injected fish died, at 5 dpc, the viral RNA was detected in liver, spleen, and head kidney from all cohabitated fish (Figure 2). The viral load peaked at 9 dpc, with the highest load of 5.38, 5.19, and 5.09 log_10_ TiLV copy/µg of total TiLV RNA, respectively, in the liver, spleen and head kidney of fish from the control diet group; thereafter, viral loads gradually declined in all TiLV-challenged groups. These viral loads from the control diet group at 9 dpc were significantly higher than those from the probiotics-supplemented groups, between which also a significant difference was observed. Importantly, viral loads from the control diet groups were never matched in the 0.5% and 1% probiotics-supplemented groups. The mean viral concentration in the liver, spleen, and head kidney of 0.5% probiotics-supplemented group was comparable to those from the control diet group. The viral loads from the 1% probiotics-supplemented group were instead always lower at all time points measured, with a more marked difference at 9 and 12 dpc. Of note, less than one log_10_ copy/µg of total TiLV RNA was detected in the organs sampled at 19 dpc from all challenged groups. No TiLV genomic RNA was detected in the sham-challenged fish and cohabitation fish during each sampling period.

### 2.3. Differential Modulation of Antiviral Response Markers during TiLV Infection

During the TiLV experimental infection, the expression profile of genes encoding for antiviral and pro-inflammatory cytokines was measured in the liver, spleen, and head kidney from the three dietaries treatment groups.

**Figure 3:**
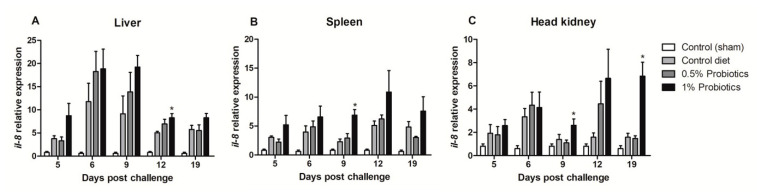
Transcription modulation of *il-8* (CXCL8) in liver (**A**), spleen (**B**), and head kidney (**C**) of fish in the control diet and 0.5% and 1% mixtures of *Bacillus* spp. probiotics-supplemented diet for 21 days before TiLV infection. Samples were collected from five fish (*n* = 5) at 5, 6, 9, 12 and 19 dpc. All treatment groups were compared to the control diet group with significant differences shown as: * *p* < 0.05.

All TiLV-challenged fish showed a consistent up-regulation of CXCL8 chemokine, also known as interleukin-8 (*il-8*), transcription from 5 to 19 dpc (Figure 3). The induction of *il-8* in the 1% probiotics-supplemented group was higher in all three internal organs, when compared to the control diet and to the 0.5% probiotics-supplemented group. *Il-8* transcription modulation was considerably higher in the liver during the infection, with some differences seen already at 5 dpc between groups (Figure 3A). A protracted higher *il-8* upregulation was seen in the 1% probiotics-supplemented group at the late stages of TiLV infection, i.e. 12 and 19 dpc.

**Figure 4:**
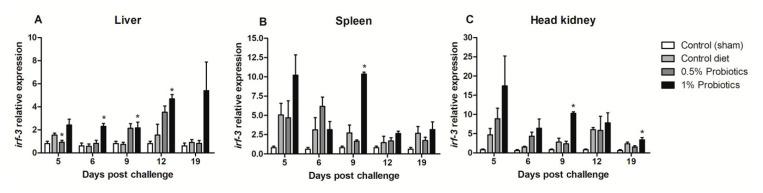
Transcription modulation of *irf-3* in liver (**A**), spleen (**B**), and head kidney (**C**) of fish in the control diet and 0.5% and 1% mixtures of *Bacillus* spp. probiotics-supplemented diet for 21 days before TiLV infection. Samples were collected from five fish (*n* = 5) at 5, 6, 9, 12 and 19 dpc. All treatment groups were compared to the control diet group with significant differences shown as: * *p* < 0.05.

The expression pattern of interferon regulatory factor 3 (*irf-3*) gene showed different induction trends between the examined organs. An early upregulation was seen in the 1% probiotics-supplemented group in spleen (Figure 4B) and head kidney (Figure 4C), at 5 and 9 dpc. *Irf-3* expression instead gradually increased from 5 to 19 dpc in the liver of 1% probiotics-supplemented fish (Figure 4A), but peaking at the later stage of TiLV infection, i.e. 12 and 19 dpc. Interestingly *irf-3* transcription was also considerably higher in probiotics-supplemented groups at 9 and 12 dpc in the liver, while at 19 dpc remained considerably higher only in the 1% probiotics-supplemented group.

**Figure 5:**
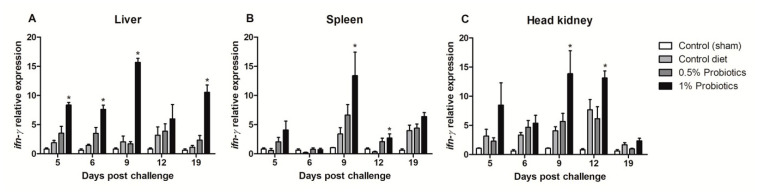
Transcription modulation of *ifn-γ* in liver (**A**), spleen (**B**), and head kidney (**C**) of fish in the control diet and 0.5% and 1% mixtures of *Bacillus* spp. probiotics-supplemented diet for 21 days before TiLV infection. Samples were collected from five fish (*n* = 5) at 5, 6, 9, 12 and 19 dpc. All treatment groups were compared to the control diet group with significant differences shown as: * *p* < 0.05.

A quicker and more sustained *ifn-γ* transcription was differentially seen in the liver and head kidney of 1% probiotics-supplemented group, although in the liver it was already significant at 5 dpc and protracted over the course of TiLV infection (Figure 5A,C). In spleen, higher transcriptional levels of *ifn-γ* were measured upon 1% probiotics supplementation, but from 9 to 19 dpc (Figure 5B). Generally, fish from the 1% probiotic-supplemented group showed the highest up-regulation of *ifn-γ*, compared to both the control diet group and also the 0.5% probiotics-supplemented group, and this difference was consistent at 9 dpc (Figure 5). 

**Figure 6:**
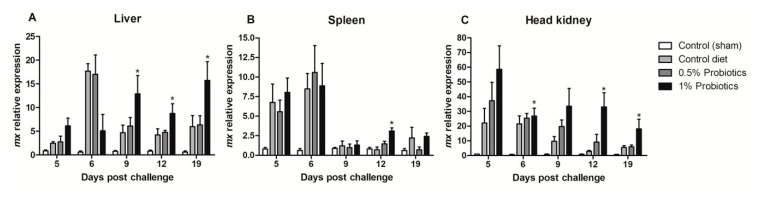
Transcription modulation of *mx* in liver (**A**), spleen (**B**), and head kidney (**C**) of fish in the control diet and 0.5% and 1% mixtures of *Bacillus* spp. probiotics-supplemented diet for 21 days before TiLV infection. Samples were collected from five fish (*n* = 5) at 5, 6, 9, 12 and 19 dpc. All treatment groups were compared to the control diet group with significant differences shown as: * *p* < 0.05.

The transcription of Mx (*mx*) gene, a key IFN-stimulated gene, showed contrasting patterns of induction (Figure 6). The higher changes were measured in the head kidney starting at 5 dpc in all infected groups but remaining selectively sustained until 19 dpc in the 1% probiotics-supplemented group (Figure 6C). In contrast, *mx* expression was selectively induced in the 1% probiotics-supplemented group from 9 to 19 dpc (Figure 6A), while, in spleen, *mx* expression was sustained in all infected groups at the early TiLV infection stages (Figure 6B).

**Figure 7:**
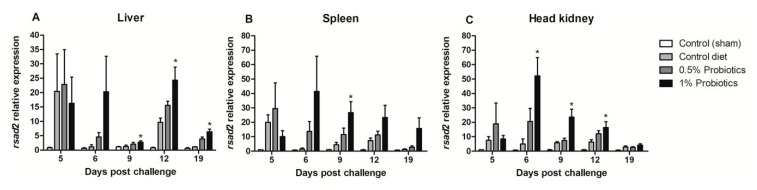
Transcription modulation of *VIPERIN* (*rsad2*) in liver (**A**), spleen (**B**), and head kidney (**C**) of fish in the control diet and 0.5% and 1% mixtures of *Bacillus* spp. probiotics-supplemented diet for 21 days before TiLV infection. Samples were collected from five fish (*n* = 5) at 5, 6, 9, 12 and 19 dpc. All treatment groups were compared to the control diet group with significant differences shown as: * *p* < 0.05.

The expression of Radical S-Adenosyl Methionine Domain Containing (*rsad*) 2 gene, also known as VIPERIN, was sustained in all infection groups, but with a higher induction pattern seen in probiotics-supplemented groups (Figure 7). A rapid up-regulation of *rsad-2* expression was detected in all organs, already at 5 dpc, peaking at 6 dpc in liver, spleen, and head kidney of fish fed 1% probiotics-supplemented diet. Thereafter, *rsad-2* expression in liver was very contained at 9 dpc, peaking at 12 dpc lowering to 19 dpc (Figure 7A). In contrast, in both spleen and head kidney *rsad-2* transcription followed by a gradual decline from 9 to 19 dpc (Figure 7B,C).

Taken together, results about the transcription of pro-inflammatory and anti-viral markers indicate that the dietary supplementation of 1% *Bacillus* spp. probiotics significantly improved the antiviral defense during the course of TiLV infection.

## 3. Discussion

TiLV is an important virus causing mass morbidities and mortalities to wild and farmed tilapia stocks worldwide [7,9,10,13]. Therefore, there is an urgent need to prevent and control TiLV disease, especially where tilapia are intensively cultured. An effective therapy or vaccine is still yet to be available to protect tilapia from TiLV infection. Probiotics have been widely used in human and farmed animals to promote general health, and successfully applied as alternatives to antibiotics [31,32]. Even in fish, several reports have shown that the dietary administration of probiotics, including live bacteria and bacteria extracts, could promote health. Probiotics may enhance fish growth performances [33], modulating digestive and antioxidant enzymes activity [34], and strengthen the innate immune response through the modulation of cytokines, innate immune cells, and the expression of genes encoding for innate defense effectors [35,36,37]. In addition, probiotics may have antagonistic effects against pathogenic microorganisms in fish [38], as shown in using Pdp11 and 51M6 (Vibrionaceae) against *Vibrio harveyi* [39]. *Bacillus* spp. showed antagonistic activity against *V. vulnificus*, *V. campbelli*, *V. parahaemolyticus* and *V. alginolyticus* promoting the growth of whiteleg shrimp (*Litopenaeus vannamei*) [40]. However, until recently, no research had demonstrated the application of probiotics to reduce the impact of TiLV infection in tilapia. To the best of our knowledge, this study is the first to evaluate the potential of *Bacillus* spp. as probiotics supplementation in fish diet and its beneficial features on tilapia health during TiLV infection.

During our study in red hybrid tilapia, despite a positive finding on the weight gain, together with lower feed conversion ratio (FCR) in the 1% probiotics-supplemented group, statistical analysis showed no difference in dietary administration of *Bacillus* spp. towards the improvement of growth parameters. Similar findings suggested that Nile tilapia (*O. niloticus*) fed with probiotics had insignificant effects on FCR improvement [41] and weight gain [41,42] during feeding trials. In contrast, a positive effect of probiotics supplementation to promote growth performance in fish was reported. Both *Lactobacillus acidophilus* and *Bacillus subtilis*-supplemented diets for 8 weeks promoted Nile tilapia growth, increased their final weight, and resulted in lower FCR [43]. Moreover, the diet supplementation with *B. cereus* var. *toyoi* for 93 days was shown to improve the growth performance of farmed rainbow trout (*Oncorhynchus mykiss*) [44]. The application of 1 × 10^5^ and 1 × 10^6^ CFU/g *B. licheniformis* to feeding, improved growth parameters in grass carp (*Ctenopharyngodon idella*) after 56 days [45]. Thus, we hypothesize that a better impact of *Bacillus* spp. probiotics on growth performances of red tilapia could have been demonstrated if supplementing probiotics for a longer time.

Interestingly, the dietary supplementation with *Bacillus* spp. significantly lowered TiLV viral load in the internal organs, including liver, spleen, and head kidney of red hybrid tilapia. This finding may be part of the explanation of the better survival of fish fed *Bacillus* spp. probiotics. In previous studies, *Bacillus* spp. had the ability to inhibit a range of pathogens, including *V. vulnificus* [46], *V. alginolyticus* [47], *A. hydrophila* [48]. Dietary supplementation with a *B. megeterium* reduced viral load in shrimp during the white spot syndrome virus (WSSV) infection [49]. Liu et al. [50] demonstrated that strains of *Bacillus* spp. isolated from different aquatic animals had strong inhibitory effects against *V. parahaemolyticus*.

To further investigate the beneficial effects of *Bacillus* spp. as probiotics dietary supplementation against TiLV infections in tilapia, we examined the transcription of a selection of pro-inflammatory cytokines and markers of the antiviral response in internal organs sampled at time points after TiLV infection. Interferon-gamma is a cytokine belonging to the fish type II IFN family, which is induced through the activation of the signal-transducing pattern recognition receptors of the innate and adaptive immune system [51]. IFN-γ expression was found to be highly induced by both bacterial and viral systemic infections, respectively, caused by *Piscine novirhabdovirus* and *Yersinia ruckeri*, in brown trout (*Salmo trutta*). During these infections, the transcription of IFN-γ was highly correlated to the bacterial and viral load in both spleen and kidney together with a strong correlation to the expression of an array of CXC chemokines, including CXCL8 or IL-8 [52,53]. Moreover, the other antiviral response markers selected for this study in red tilapia, including *irf-3*, Mx, and VIPERIN (or *rsad-2*), were previously found to be strongly induced during several viral infections in other teleosts [54,55,56] and involved in the IFN response in tilapia [57]. Previously, studies showed that the administration of probiotics could modulate the expression of pro-inflammatory cytokines, such as *ifn-γ*, useful for activating the cell-mediated immunity, enhancing antigen presentation, and leading to inhibiting pathogen replication [35,58,59]. Probiotics can also enhance the expression of CXCL8 or *il-8*, which is one of the best-studied chemokines in fish, known to attract and activate neutrophils, and to be induced by a range of pathogens [60,61,62,63]. Our study demonstrates that the expression of *ifn-γ* and *il-8* was significantly upregulated in the liver, spleen, and head kidney in the group fed 1% of *Bacillus* spp. probiotics-supplemented diet. There is a possibility that the capability of *Bacillus* spp. to induce *ifn-γ* and *il-8* in immune cells subsequently contributed to improving the antiviral response mounted by the host against TiLV. Likewise, an upregulation of *ifn-γ* upregulation was found in Goldfish (*Carassius auratus)* fed *B*. *velenzensis*-supplemented diet [64]. Moreover, dietary supplementation with *Lactococcus lactis* increased *ifn-γ* expression in olive flounder during *Streptococcus iniae* infection [65]. The master regulators of the interferon pathway, including *irf-3* and *irf-7*, play a critical role in triggering *ifn-γ* activation, thus leading to the activation of interferon stimulate genes to produce antiviral effectors, such as MX proteins and RSAD-2 that inhibit viral polymerases in the nucleus and limit viral replications [66,67]. In this present study, the expression of *irf-3* was upregulated in red tilapia fed *Bacillus* spp. at 0.5% and 1% diets. Moreover, *mx* and *rsad2* were found to be significantly upregulated in the probiotics-supplemented group during TiLV infection. The administration of *B. subtilis* dietary supplementation at (10^9^ CFU kg/diet) was shown to significantly increase *mx* expression in the head kidney of Asian seabass (*Lates calcarifer*) after 24 h of probiotics feeding [68]. Another probiotic bacteria, *Vagococcus fluvialis*, also stimulated *mx* expression in the head kidney of European sea bass (*Dicentrarchus labrax*) [69]. Despite these pieces of transcriptional modulation evidence, further studies are still needed to better understand the exact mechanisms of probiotics on the activation of tilapia immune responses during TiLV infection.

Probiotics can boost and support the natural antimicrobial resistance of fish, leading to better survival against pathogen challenge [31]. Liu et al. [70] demonstrated the resistance of Nile tilapia against *S. agalactiae* after 8 weeks of feeding on a diet containing *B. subtilis* at 10^8^ CFU/g. For viral infections, a diet containing *S. cerevisiae* at the concentration of 5.3 × 10^7^ CFU/kg increased the survival of groupers against iridovirus and *Streptococcus* spp. at the end of a 4 week trial [71]. A similar study showed that the application of *Lactobacillus plantarum* at 10^8^ CFU/kg for 4 weeks increased the survival of orange-spotted grouper during *Iridovirus* infection [72]. Likewise, hybrid Hulong groupers *(E. fuscoguttatus* × *E. lanceolatus*) fed *B. subtilis* at 1 × 10^8^ and 1 × 10^10^ CFU/g diet had lower mortality during *Iridovirus* infection [28]. Feeding 0.5% and 1% of 2.4 × 10^8^ CFU/g Lactobacil and Sprolac to olive flounder for a period of 30 days significantly reduced mortality during the lymphocystis disease virus [27]. In this study, lower mortality during TiLV infection was found in tilapia fed 0.5% and 1% *Bacillus* spp.-supplemented diets, compared to the control diet group. To the extent of our knowledge, the present study is the first report of the effect of *Bacillus* spp. probiotic against TiLV infection.

## 4. Conclusions

In summary, our study demonstrates that an oral administration of *Bacillus* spp. may have beneficial antiviral effects against TiLV infection in red hybrid tilapia. The preventive oral administration of probiotic diet increased the survival of tilapia upon TiLV infection. The host innate immune response was improved, as indicated by the consistently increased transcription of a pro-inflammatory chemokine and antiviral markers in probiotics-supplemented groups, and with a higher effect in the group supplement with 1% of *Bacillus* spp. probiotics. This pilot study will inform the adoption of prevention strategies aimed at the control of TiLV infections, opening to further studies on the health management using probiotics to strengthen the fish immunity against TiLV and other relevant infectious agents.

## 5. Materials and Methods

### 5.1. Experimental Fish Source and Use

Seven hundred red hybrid tilapia (total body weight (TBW) 10 ± 0.5 g) were transferred from a tilapia hatchery certified with no history of TiLV in Saraburi province, Thailand, to aquarium facility at the Faculty of Veterinary Medicine, Kasetsart University, Thailand. Fish were acclimated for 2 weeks in a 150 L tank, with the following water quality parameters: 5.54 ± 0.21 mg/L dissolved oxygen, 0.2 ± 0.05 mg/L ammonia, 0.1 ± 0.03 mg/L nitrite, pH 7.2 ± 0.24. Water temperature was maintained at 28 °C, and the photoperiod was kept with 12 h light/12 h dark. Fish were fed commercial feed (INVE Technologies Co., Ltd., Nonthaburi, Thailand) at the rate of 3% of TBW/day, and abnormal behaviors and daily mortality were recorded. Prior to the start of the experiment, five fish were randomly sampled and examined for the presence of any ecto- and endo-parasite, using gill mucus, skin, and gut smears checked under light microscopy. The head kidney and spleen were screened for bacterial infections, aseptically streaked onto TSA plates and growth at 30 °C for 48 h. The head kidney and spleen were screened for TiLV infection by RT-qPCR, using the protocol previously described [73] (primers in Table 2). The animal use protocol was reviewed and approved by the Kasetsart University Animal Use Committee, under the protocol number ACKU63-VET-017.

### 5.2. Experimental Diet and Feeding

**Figure 8:**
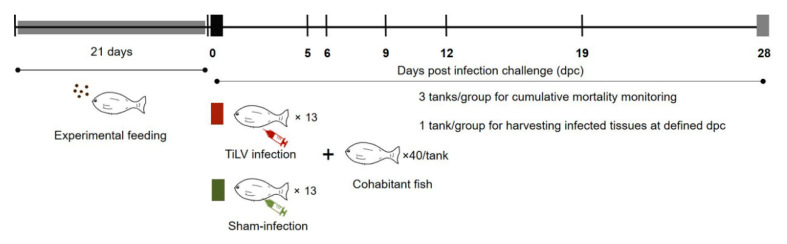
Experimental design. Fish were fed a commercial diet or probiotics-supplemented diet for 21 days. Thereafter, experimental fish were cohabited with TiLV IP-infected vectors (clipped pelvic fin) and tissue samples (*n* = 5 per group) were collected at 5, 6, 9, 12 and 19 days post infection challenge (dpc). Three replicate tanks were set for cumulative mortality analysis and TiLV quantification, while fish from an additional tank were sampled for the analysis of immune genes transcription modulation.

A commercial mixture of *Bacillus subtilis*, *B. licheniformis*, and *B. pumilus* (Secure Yield) was provided by INVE Technologies Co., Ltd., Dendermonde, Belgium. A 1.8 mm tilapia diet, with >32% crude protein, >4% crude fat, was coated with the mixture of *Bacillus* spp. to reach final concentrations of 1 and 2 × 10^7^ CFU/g. A total of 480 red hybrid tilapia were randomly distributed to 12 tanks (150 L), allocating 40 fish/tank. Three experimental groups were defined: (1) control diet; (2) fed supplemented with 0.5% probiotic; and (3) fed supplemented with 1% probiotic. For each experimental group, one tank was designed as the sampling tank, used for harvesting tissue samples at defined time points, while the other three tanks were monitored to record the cumulative mortality until the experiment termination (mortality tanks). Fish were fed with control and probiotic-supplemented feed for 21 days prior to any experimental challenge (Figure 8). Additionally, a group of 40 fish was maintained as an untreated control (sentinel tank) for the entire experimental period and fed the control diet. During the whole experiment, fish were fed at a daily rate of 3% TBW, according to each experimental diet.

### 5.3. TiLV Source

The TiLV strain (VETKU-TV01) was isolated from infected red hybrid tilapia in Pathum Thani province, Thailand in 2016 [30]. TiLV was propagated in snakehead fish (*Ophiocephalus striatus*) E-11 cells [74]. The E-11 cells were purchased from the European Collection of Authenticated Cell Cultures (ECACC #01110916) and maintained in L-15 Leibovitz (Sigma, Cambridge, MA, USA) medium supplemented with foetal bovine serum (Gibco, Paisley, UK) and L-glutamine at 25 °C, without CO_2_, and without the addition of antibiotics. The titer of TiLV (TCID_50_/_mL_) was determined following the protocol described by Reed and Muench [75]. The cell suspension was centrifuged at 3000× *g* for 10 min and the supernatant was stored at −80 °C until the challenge test.

### 5.4. TiLV Infection Challenge

Red hybrid tilapia were anesthetized with eugenol (Aquanes^®^, Better Pharma, Thailand) at a concentration of 3 mL/L for 5 min prior to be intraperitoneally injected (IP) with 50 µL of viral suspension in L-15 medium, at the concentration of 10^5^ TCID_50_/_mL_. The TiLV IP-challenged fish were clipped at the pelvic fin and allocated to each experimental tank for cohabitation with previously unexposed fish (with unclipped pelvic fin) at a ratio of 1:3 at 0 dpc. For the sham-infection group, 13 fish were IP-injected using 50 µL of L-15 medium suspension collected from uninfected E-11 cells, and allocated to the sentinel tank at 0 dpc. The experimental design is illustrated in Figure 8.

Water quality parameters were maintained at 0.25 ± 0.63 mg/L ammonia, 0.1 ± 0.05 mg/L nitrite, 4.92 ± 0.57 mg/L of dissolved oxygen, pH 7.2 ± 0.03, and water temperature kept at 28 ºC. The appearance of any clinical sign and occurrence of mortality were recorded daily until 28 dpc using the tanks designed for mortality recording. At 5, 6, 9, 12, and 19 dpc, five fish with unclipped pelvic fin were randomly sampled from the sampling tank of each treatment group and from the sentinel tank after 21 days feeding. Fish were sacrificed using eugenol (Aquanes^®^, Better Pharma, Bangkok, Thailand) solution at a concentration of 3 mg/L. Liver, head kidney, and spleen were aseptically dissected and stored at −20 °C until the analysis was performed.

### 5.5. Growth Performances

The experimental fish (*n* = 25 from each tank) were weighed at the beginning prior to the feeding experiment (initial body weight), and at 21 days before the challenge study (final body weight). Weight gain (WG) average daily gain (ADG), feed conversion ratio (FCR) and feed efficiency (FE) were determined as follows.

WG = [final body weight (g) − initial body weight(g)] ÷ initial body weight(g)
Feed efficiency (FE) = [weight gain(g) ÷ amount of ingested feed (g)]
FCR = feed intake ÷ weight gain
ADG (g) = weight gain ÷ number of days on feed

### 5.6. Total RNA Extraction and cDNA Synthesis

Total RNA was extracted from sampled tissues at the defined experimental time points using Trizol^®^ solution (Invitrogen, Waltham, MA, USA), following the manufacturer’s instructions. RNA quality and concentration were estimated using spectrophotometry (NanoDrop 2000C, Thermoscientific, USA). One µg of total RNA was reverse transcribed using ReverTraAce^®^ kit (Toyobo, Japan), following the manufacturer’s instructions. The resulting cDNA was diluted with TE buffer pH 8.0 and stored at −80 °C.

### 5.7. Viral Load Quantification

The amount of TiLV genomic RNA in liver, spleen, and head kidney of control and probiotic supplementation fish was analyzed by an RT-qPCR assay as previously described [73,76]. The assays were performed in the CFX96™ thermocycler (BioRad, Hercules, CA, USA). The load of TiLV genomic RNA was calculated from the standard curve of ten-fold serial dilutions of plasmid containing TiLV segment 3, as previously described [76].

### 5.8. Gene Expression Analysis by RT-qPCR

RT-qPCR was performed using a MicroAmp Optical 96-well reaction plate (BioRad) in a 10 µL reaction: consisting of 5 µL iTaq^™^ universal SYBR green supermix (BioRad), 0.3 µL of each forward and reverse primer, 0.4 µL of molecular grade water, and 4 µL of cDNA template. All samples were run in triplicate. General cycling parameters were set as follows: denaturation at 95 °C for 3 min, followed by 40 cycles of 95 °C for 10 s, and 60 °C for 30 s. At the end of the qPCR cycle, samples were subjected to melting curve analysis at a temperature ranging from 65 to 95 °C with 0.5 °C per 5 s increments. RT-qPCR assays were performed using a CFX96 Touch™ machine (BioRad). Primer pairs used in this experiment are listed in Table 2. RT-qPCR data were retrieved using CFX Maestro™ software (Biorad). Differences in the Ct (ΔCt) values of immune genes and β-actin gene were calculated. The difference of ΔCt for the probiotic-supplemented samples and control samples was expressed as (ΔΔCt) value that allowed for measurement of the change in the expression of immune-related genes [77].

### 5.9. Statistical Analysis

Data retrieved from growth performance, viral load, and the expression of immune-related genes were expressed as mean ± standard error of mean in control and treatment groups. An ordinary one-way ANOVA with Tukey’s multiple comparisons test, using GraphPad Prism software 5.0.1 (GraphPad Inc., La Jolla, CA, USA), was applied to the statistically significant difference at *p* < 0.05, where the different amounts of experimental probiotic diets were used as an explanatory variable. Cumulative mortality significance was set at *p* < 0.05 during the TiLV infection.

## Figures and Tables

**Table 1:** Growth performance of red hybrid tilapia (*n* = 25) fed with a control diet and 0.5% and 1% mixtures of *Bacillus* spp. probiotics-supplemented diet for 21 days.

Parameters	Treatments		
	Control Diet	0.5% Probiotics	1% Probiotics
Initial weight (g)	11.4 ± 0.82	11.3 ± 1.04	9.9 ± 0.20
Final weight (g)	24.9 ± 0.99	25.0 ± 0.78	24.2 ± 0.24
Weight gain (g)	13.6 ± 0.18	13.7 ± 0.61	14.3 ± 0.10
Weight gain (%)	218.1 ± 7.59	220.2 ± 15.65	242.1 ± 2.79
Feed efficiency	0.97 ± 0.01	0.98 ± 0.04	1.02 ± 0.007
Average daily gain (ADG) (g)	0.65 ± 0.009	0.65 ± 0.02	0.68 ± 0.004
Feed conversion ratio (FCR)	1.03 ± 0.01	1.02 ± 0.04	0.98 ± 0.006

**Table 2:** List of primers used for RT-qPCR in red hybrid tilapia.

Gene	Primer Sequence (5′-3′)	Amplicon Size (bp)	Accession Number	Source
β-actin	F: GTGGGTATGGGTCAGAAAGAC	111	XM003443127	Mugimba et al., 2019
	R: GTCATCCCAGTTGGTCACAATA			
TiLV	F: CTGAGCTAAAGAGGCAATATGGATT	112	KU751816	Tattiyapong et al., 2018
	R: CGTGCGTACTCGTTCAGTATAAGTTCT			
*il-8*	F: TCGCCACCTGTGAAGGCA	116	NM001279704	*this study*
	R: GCAGTGGGAGTTGGGAAGAAT			
*Irf-3*	F: CTGTGTTTTCGGAATCTGCTG	182	XM005448320	*this study*
	R: CATTACTGGATACTGCTGTTGC			
*ifn-γ*	F: GAAACTTCTGCAGGGATTGG	132	NM001287402	Velázquez et al., 2017
	R: CTCTGGATCTTGATTTCGGG			
*Mx*	F: ACCCTTGAGCTGGTGAATCA	174	XM003442686	Velázquez et al., 2017
	R: ATCCTGAGTGAATGCGGTCA			
*RSAD-2*	F: ATCAACTTCTCTGGCGGA	161	XM003453237	*this study*
	R: AGATAGACACCATATTTCTGGAAC			
